# Growth factor stimulation of cardiomyocytes induces changes in the transcriptional contents of secreted exosomes

**DOI:** 10.3402/jev.v2i0.20167

**Published:** 2013-05-17

**Authors:** Nina Gennebäck, Urban Hellman, Linus Malm, Göran Larsson, Gunnar Ronquist, Anders Waldenström, Stellan Mörner

**Affiliations:** 1 Department of Public Health and Clinical Medicine, Umeå University, Umeå, Sweden; 2 Department of Cardiology, Heart Centre, Umeå University, Umeå, Sweden; 3 Department of Medical Biochemistry and Biophysics, Umeå University, Umeå, Sweden; 4 Department of Medical Sciences, Clinical Chemistry, University Hospital of Uppsala, Uppsala, Sweden

**Keywords:** extracellular vesicles, exosomes, cardiomyocytes, growth factors, gene expression

## Abstract

Exosomes are nano-sized extracellular vesicles, released from various cells, which can stimulate or repress responses in targets cells. We recently reported that cultured cardiomyocytes are able to release exosomes and that they, in turn, are involved in facilitating events in target cells by alteration of gene expression. We investigated whether external stimuli of the cardiomyocyte might influence the transcriptional content of the released exosomes.

Exosomes were isolated from media collected from cultured cardiomyocytes (HL-1) with or without growth factor treatment (TGF-β2 and PDGF-BB), with a series of differential centrifugations, including preparative ultracentrifugation and separation with a sucrose gradient. The exosomes were characterized with dynamic light scattering (DLS), electron microscopy (EM) and Western blot and analyzed with Illumina whole genome microarray gene expression.

The exosomes were rounded in shape and had an average size of 50–90 nm in diameter with no difference between treatment groups. Analysis of the mRNA content in repeated experiments conclusively revealed 505 transcripts in the control group, 562 in the TGF-β2-treated group and 300 in the PDGF-BB-treated group. Common transcripts (217) were found in all 3 groups.

We show that the mode of stimulation of parental cells affects the characteristics of exosomes released. Hence, there is a difference in mRNA content between exosomes derived from cultured cardiomyocytes stimulated, or not stimulated, with growth factors. We also conclude that all exosomes contain a basic package consisting of ribosomal transcripts and mRNAs coding for proteins with functions within the energy supply system.

Exosomes are extracellular nano-sized vesicles, generated in multivesicular bodies (MVB) inside the cell and released via exocytosis by the fusion of MVB to the plasma membrane (1, 2). They have pleiotropic functions such as antigen presentation (3) and intercellular transfer of proteins (4) and nucleic acids (5–7). Accordingly, they are involved in cell-to-cell communication (8) and are released from a wide range of cells, for example, T-cells (9), tumour cells (10–12), mast cells (13), prostatic acinar cells (14) and neuronal cells (15). Exosomes from different cellular origins harbour a common set of molecules that are representative of their biogenesis, structure and trafficking commission. In addition, they contain cell-type specific components that correspond to the specific biological function of the parental cell.

In 2007, Gupta et al. were the first to show that cardiomyocytes release exosomes (16). They showed that heat shock proteins released from the heart could be found in cardiomyocyte exosomes. This finding opens up a new field in cardiology where exosomes are proposed as a new way of communication within the heart and between other organs. We recently demonstrated that cultured cardiomyocytes release exosomes containing DNA and RNA and that they, in turn, are involved in facilitating events in target cells by alteration of gene expression (7).

To further investigate our finding, the cultured cardiomyocytes were stimulated with growth factors and the RNA content of the released exosomes were studied to determine if the stimuli changed the RNA content or not, thereby proposing that the external milieu is reflected in differences in exosomal signalling. We have observed in studies on rat that TGF-β2 (transforming growth factor-β2) is highly involved in both chronic and acute hypertrophy (17). TGF-β2 was therefore a natural choice of growth factor. PDGF-BB (platelet-derived growth factor BB) was chosen as it belongs to another family of growth factors than TGF-β2 but has still been associated with cardiac hypertrophy.

TGF-β2 is a pleiotropic cytokine, meaning that it has an effect on many different types of cells, and is a member of the transforming growth factor family. TGF-β2 is also required for valve remodelling during cardiac development (18) and it has been demonstrated that TGF-β2 enhances the differentiation of cardiomyocytes from embryonic stem cells (19). In cardiac hypertrophy, TGF-β2 regulates cardiac remodelling (20) and alters the gene expression and thereby promotes the foetal gene programme (21).

Cardiomyocytes express 2 types of PDGF receptors to which PDGF-BB has affinity. PDGF-BB is known to promote hypertrophy and an anti-apoptotic effect in target cells (22, 23), and the expression of PDGF-BB rises as a response to stress and pressure overload (24).

In this study, we treated cultured cardiomyocytes with TGF-β2 and PDGF-BB, to study if the characteristics of the exosomes, including their possible messenger function, are affected by the external milieu of the cardiomyocytes.

## Materials and methods

### Cell culture

HL-1 is a cell line derived from adult mouse heart, displaying phenotypic features typical of adult cardiomyocytes (25). This cell line was acquired from Dr. W.C. Claycomb (Louisiana State University Medical Center, New Orleans, LA). Cardiomyocytes were grown in T-75 culture flasks coated with fibronectin (Sigma-Aldrich)-gelatin (Fisher Scientific) and maintained in Claycomb Medium (JRH. Biosciences) supplemented with ultracentrifuged 10% foetal bovine serum (JRH. Biosciences), 0.1 mM norepinephrine (Sigma-Aldrich) (in a solution of 30 mM ascorbic acid), 2 mM L-glutamine (Life Technologies), 100 U/mL penicillin and 100 µg/mL streptomycin (Life Technologies). During culture, the medium was changed every 24 h. The cells were passaged at confluence (approximately every third day) by trypsinization (Trypsin- EDTA, Life Technologies). All culture flasks were kept in an atmosphere of 95% air to 5% CO_2_, 37°C and at a relative humidity of approximately 95%.

### Pre-treatment of cells

In the treatment groups, the cells were stimulated with either TGF-β2 (10 ng/mL) and PDGF-BB (100 ng/mL; Biosource, Invitrogen) dissolved in serum-free and antibiotic-free media. Flasks with no growth factor addition were used as controls. After 23 h, the medium from each growth condition was collected and frozen before exosome isolation.

### Cell harvest

Cells were harvested with trypsin, after collection of the cell culture media, and washed twice with PBS (phosphate-buffered saline). They were again suspended in PBS and stored at −80°C. After lysation of the cell suspension with a lysis buffer, the protein concentration was measured with a BCA protein assay kit (Pierce Protein Biology Products, Rockford, IL, USA).

### Exosome isolation

Differential centrifugations were carried out of the collected cell culture media to obtain exosomes; centrifugation for 30 min at 2,000×*g* and 35 min at 10,000×*g*, after which the supernatant was filtered through a 0.22-µm filter. The exosomes were then pelleted by ultracentrifugation of the filtrate at 110,000×*g* for 2 h. Pellets were re-suspended in PBS. A sucrose gradient was prepared by 10 mL each of 40 and 20% sucrose, respectively. One mL of resuspended exosomes was loaded onto the top of the gradient, followed by immediate ultracentrifugation at 110,000×*g* for 2 h. After centrifugation, 2 mL fractions were collected from the band of the gradient. The fractions were washed with PBS and ultracentrifuged at 110,000×*g* for 2 h. The pellets were resuspended in PBS. All ultracentrifugations were performed using the L-90 Beckman centrifuge (Beckman Instruments, Inc., Fullerton, CA) and the SW-41 rotor (Beckman Instruments, Inc., Fullerton, CA) at 4°C.

### Western blot

Following lysation of isolated exosomes from 10 T75 culture flasks/group and cells with a lysis buffer, the protein concentration was measured with a BCA protein assay kit (Pierce Protein Biology Products, Rockford, IL) to establish that the same amount of protein was used in the analysis. The lysates of the exosome samples and the cell suspension were separated by 10% SDS-PAGE. Protein was transferred to a polyvinylidene difluoride membrane using a Bio-Rad Mini Transblot electrophoretic transfer cell, according to the manufacturer's protocol. The blot was blocked with 5% bovine serum albumin (BSA) in Tris-buffered saline (TBS) containing 0.1% (v/v) Tween 20 (TBST). Mouse monoclonal anti-TSG101 (abcam, UK), rabbit polyclonal anti-GRP78 (abcam, UK) and mouse monocolonal anti-CD63 (BD Pharmingen, USA) were used as primary antibodies in the different experiments according to the manufacturer's protocols. CD63 (a tetraspanin) is a common exosome marker (26), TSG101 (tumour susceptibility gene protein 101) is another exosome marker that is also present in cell suspension (26) and GRP78, a member of the heat shock protein family of molecular chaperons, is a protein associated with the endoplasmatic reticulum and cellular stress and found in apoptotic bodies (27). Polyclonal rabbit anti-mouse or anti-rabbit IgG-horseradish peroxidase (HRP) secondary antibodies (Dako, Denmark) were added for 1 h. After extensive washes with TBS, spots were detected using ECL Advance Western Blotting Detection Kit (GE Healthcare Life sciences, UK). All Western blot experimental results were scanned using Molecular Imager^®^ GelDoc™ (Biorad, CA).

### Dynamic light scattering size analysis of the exosomes

The exosome size/diameter was estimated by dynamic light scattering (DLS). The mean hydrodynamic diameter of exosomes was calculated by fitting a Gaussian function to the measured size distribution.

Prior to the DLS measurements, each exosome sample (4 controls, 4 TGF-β2 and 3 PDGF-BB-treated samples, the same samples used in the microarray analysis) was shaken at 4°C for 20 min to dissolve possible exosome aggregates. About 50 µL of each sample was added to a cuvette with a 10 mm path length. DLS measurements were conducted at 20°C using a Nano Zetasizer (Malvern Instruments Ltd., UK) operating at 633 nm and recording the back scattered light at an angle of 173°. The sample temperature was allowed to equilibrate for 10 min before each measurement. The light scattering was recorded for 200 s with 10 replicate measurements. DLS signal intensity was transformed to volume distribution [volume (%)], assuming a spherical shape of the exosomes, using the Dispersion Technology Software v.5.10 (Malvern Instruments Ltd., UK). The peak maximum of the Gaussian function was used to estimate exosome size. Gaussian fitting, mean value and standard deviation were calculated and compared using OriginPro 9.0.0 (OriginLab Corp, USA). Peak maximum of the Gaussian function was used to estimate exosome size. Outliers were detected and rejected by calculation of the Dixon's *Q* ratio, using p=0.05 (28).

### Electron microscopy

One pooled sample of exosomes, including isolated exosomes from 10 T75 culture flasks from each exosome group, that is, control, TGF-β2 treated and PDGF-BB treated, were, after isolation, washed 2 extra times with PBS at 110,000×*g* for 2 h to remove excess sucrose. Exosomes were then resuspended in 10 mM Tris-HCl buffer (pH 7.4) with 10 mM MgCl_2_, after which 4 µL of the suspension was allowed to adhere to Formvar-coated grids for 3 min at room temperature. The samples were negatively stained with 1% sodiumsilicotungstate. The sample grids were examined in a JEOL 1,230 TEM (JEOL Scandinavia local office, Sollentuna, Sweden) and digital images were captured by using a Gatan MSC 600CW (Gatan Inc., Abingdon, UK).

### RNA preparation

For each exosome sample (1 T75 culture flask), total RNA was isolated using the RNeasy Fibrous Tissue Kit (Qiagen AB Nordic, Sollentuna, Sweden). The concentrations of the RNA were measured in a NanoDrop ND-1,000 Spectrophotometer (NanoDrop Technologies Inc., Wilmington, DE). The integrity of the RNA was analyzed with a 2,100 Bioanalyser (Agilent Technologies Inc., Palo Alto, CA).

Total RNA was also isolated from the corresponding parental cells of each exosome sample. This was also done using the RNeasy Fibrous Tissue Kit (Qiagen AB Nordic, Sollentuna, Sweden). The concentration of the RNA was measured in a NanoDrop ND-1,000 Spectrophotometer (NanoDrop Technologies Inc., Wilmington, DE).

### Microarray gene expression

The Illumina Totalprep RNA Amplification Kit (Ambion, Carlsbad, CA) was used to convert aliquots of total RNA to biotinylated double-stranded cRNA for both the exosome RNA samples and the corresponding parental cell RNA samples. The biotinylated cRNA samples were hybridized to MouseRef-8 Expression Beadchip (Illumina Inc., San Diego, CA), incubated with streptavidin-Cy3 and scanned using the Illumina Beadstation GX, analyzing 18,139 gene transcripts. Four samples in each exosome group and 4 samples in each corresponding cell group were analyzed. Each sample consisted of exosomes or cells isolated from 1 culture flask, rendering results from 4 different culture flasks/experiments in each exosome or cell group. The data from the exosome microarray have the GSE40503 accession number in the gene expression omnibus (GEO) and the corresponding cell data have the GSE45207 accession number. The significant transcript can also be found in Vesiclepedia (29).

### Data analysis of gene expression microarrays

To detect exosome mRNA content, microarray data were analyzed using the gene expression module in Illumina Beadstudio software, version 3.3.7.

In each exosome group (control, TGF-β2- and PDGF-BB-treated), 4 samples were analyzed. Intensity data were normalized using the Beadstudio cubic spline algorithm. Detection p-value <0.01 was used to filter which gene transcripts that had significantly detected signals on the microarrays and therefore represented mRNA assignable to the exosomes.

In each corresponding cell group (control, TGF-β2- and PDGF-BB-treated), 4 samples were analyzed. Intensity data were also normalized using the Beadstudio cubic spline algorithm. Detection p-value <0.05 and a signal intensity >30 were used to filter which gene transcripts that had significantly detected signals on the microarrays and therefore represented mRNA assignable to the cells.

MetaCore™ (GeneGo Inc., USA) was used to find common transcriptional relations between the growth factor added to the cardiomyocytes and the transcripts found in the derived exosomes and this was done with a direct interaction network. Significant transcripts specifically found in the 2 exosome groups derived from growth factor stimulated cardiomyocytes were included in this analysis. These transcripts were not found in the control exosome group.

## Results

### Western blot

**Fig. 1:**
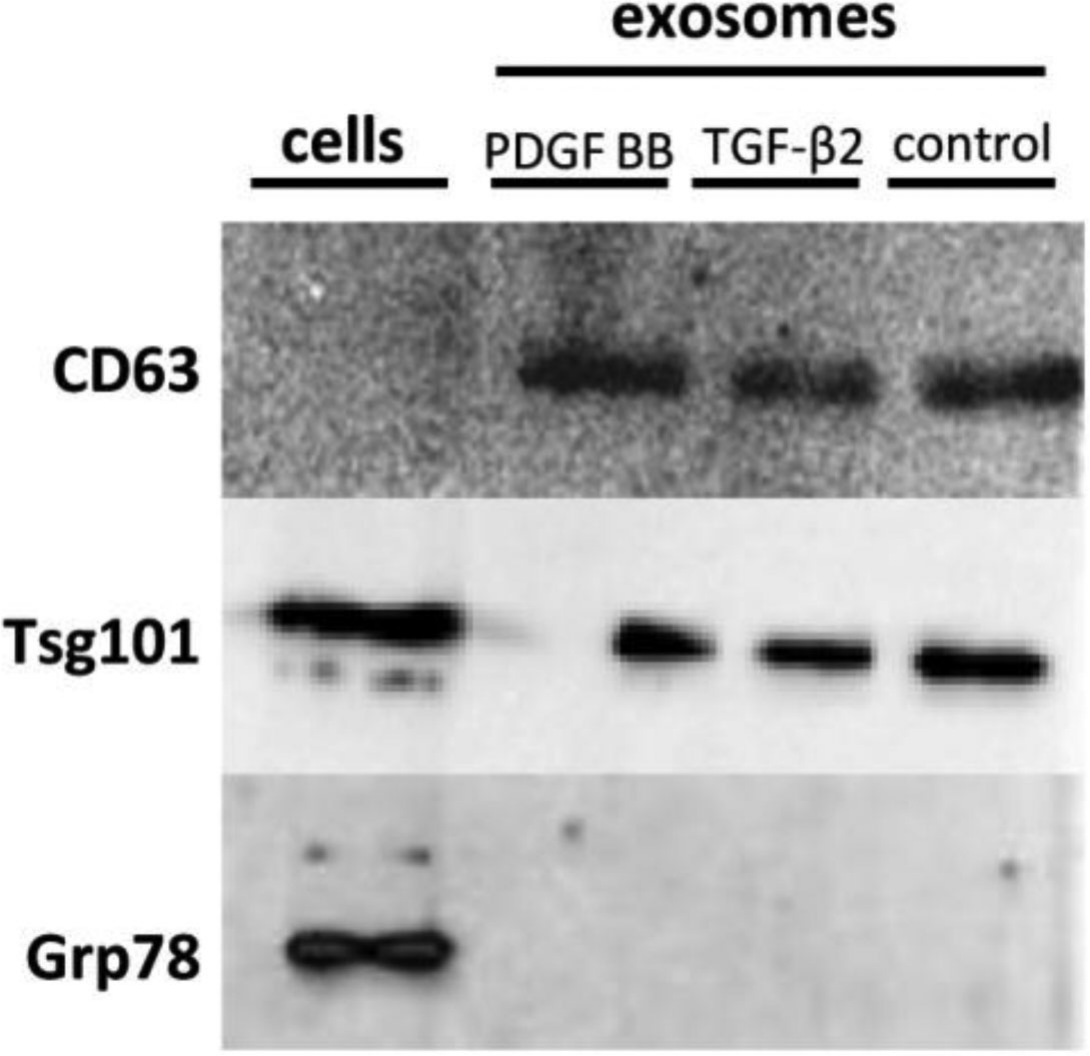
Western blots results. CD63 is present in all 3 of the exosome samples but not in the cell suspension, while Tsg101 is enriched in both the exosome samples and the cell suspension. Grp78 is only found in the cell suspension, indicating no detectable contamination of apoptotic bodies. These results demonstrate the authenticity of the exosome samples.

CD63 was recognized in exosomes of the control group as well as in the 2 treatment groups, but not in the cell suspension, indicating that exosomes had been successfully purified (Fig. 1). TSG101 was found both in the exosome samples and the cell suspension (Fig. 1). GRP78 was only found in the cell suspension and not in the exosome samples (Fig. 1), indicating no detectable contamination of apoptotic bodies. No sensitivity problems with antibodies used were apparent.

### Dynamic light scattering size analysis of the exosomes

**Fig. 2:**
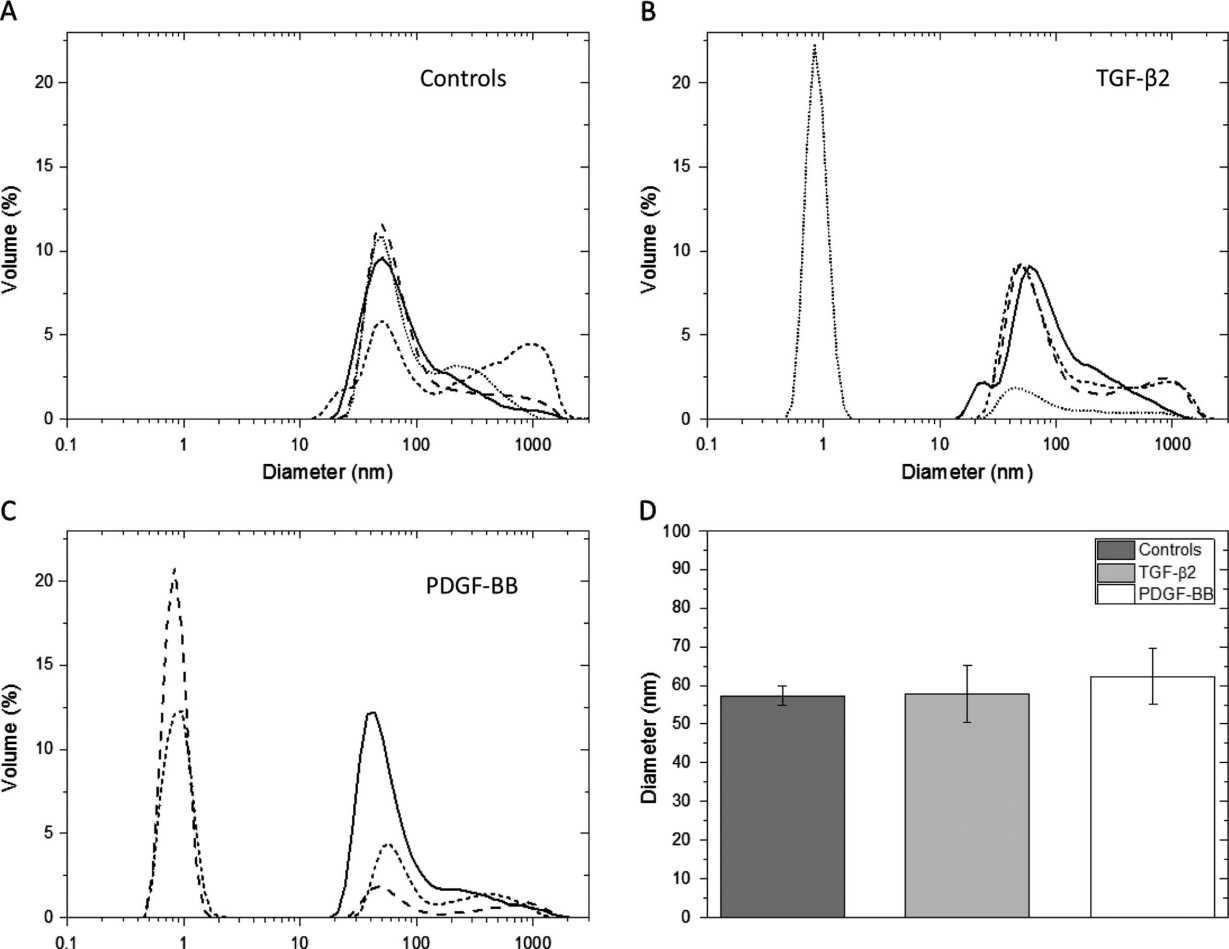
Dynamic light scattering, size determining results. DLS measurements of exosomes released from cells after 23 h incubation of controls (A), cells stimulated with TGF-β2 (B) and cells stimulated with PDGF-BB (C). The DLS showed an average size between 57.3, 57.8 and 62.3 nm for controls, TGF-β2- and PDGF-BB-treated samples, respectively (D). No significant intergroup differences are seen between the groups. A peak corresponding to a hydrodynamic radius of approximately 1 nm was seen in some samples (B and C), which is most likely a contamination of residual sucrose left after the sucrose gradient centrifugation. Larger elements, over 100 nm in size, were also observed (A–C). Such elements are probably aggregates of exosomes also present in the electron microscopy analysis.

The DLS measurement showed an average exosome size between 57 and 72 nm in diameter after 23 h incubation, with no intergroup differences (control and the 2 treatment groups) (Fig. 2). A peak corresponding to a diameter of approximately 1 nm was also seen in some samples (Fig. 2B and C). This is most likely a contamination of residual sucrose left after the sucrose gradient centrifugation. Larger elements, over 100 nm in size, were present (Fig. 2A–C). Such elements might be aggregates of exosomes also observed in the EM analysis.

### Electron microscopy

**Fig. 3:**
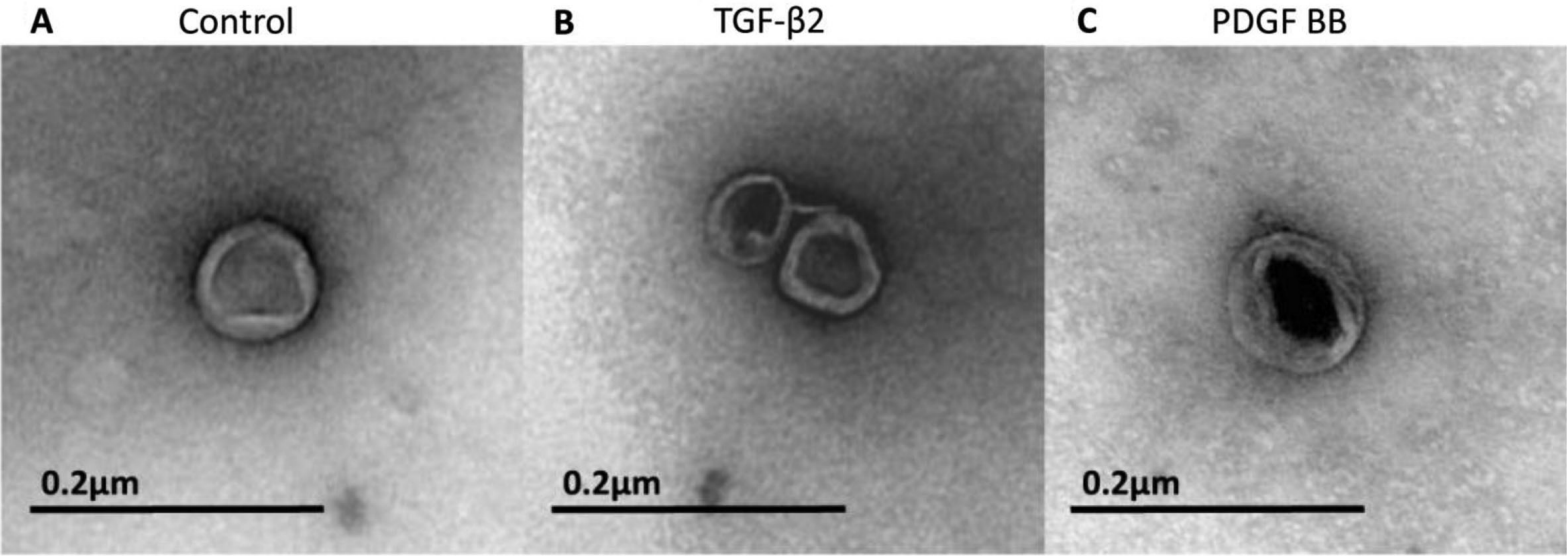
Electron microscopy of purified exosomes. The exosomes are rounded and surrounded by a lipid bilayer. They have a somewhat electron dense content displaying a similar size of approximately 50–90 nm in diameter in all 3 treatment groups. A) Example of an exosome in the control group. B) Example of 2 exosomes in the TGF-β2-treated group. C) Example of an exosome in the PDGF-BB-treated group. All 3 exosome groups contained exosomes with different densities. Shown in the figure are the best pictures of exosomes from each group that happened to be of different density, but this does not indicate that there were any differences between the groups when taking all exosomes into account.

EM analysis of the isolated exosomes revealed the presence of vesicles with a characteristic rounded shape and with a diameter ranging from 40 to 90 nm (Fig. 3 A–C). No differences in size or appearance were found between the control exosomes and the exosomes derived from growth factor treated cardiomyocytes (Fig. 3A–C). All 3 exosome groups consisted of exosomes with varying electron densities. Large aggregates of exosomes were occasionally found.

### Microarray gene expression

The Bioanalyzer analysis showed no signs of ribosomal 18S or 28S peaks, indicating that the mRNA present has originated from the exosomes and not from cell suspension or debris. One sample was excluded from the PDGF-BB group since it was recognized as an outlier due to poor hybridization and labelling.

### Data analysis of gene expression microarrays

**Fig. 4:**
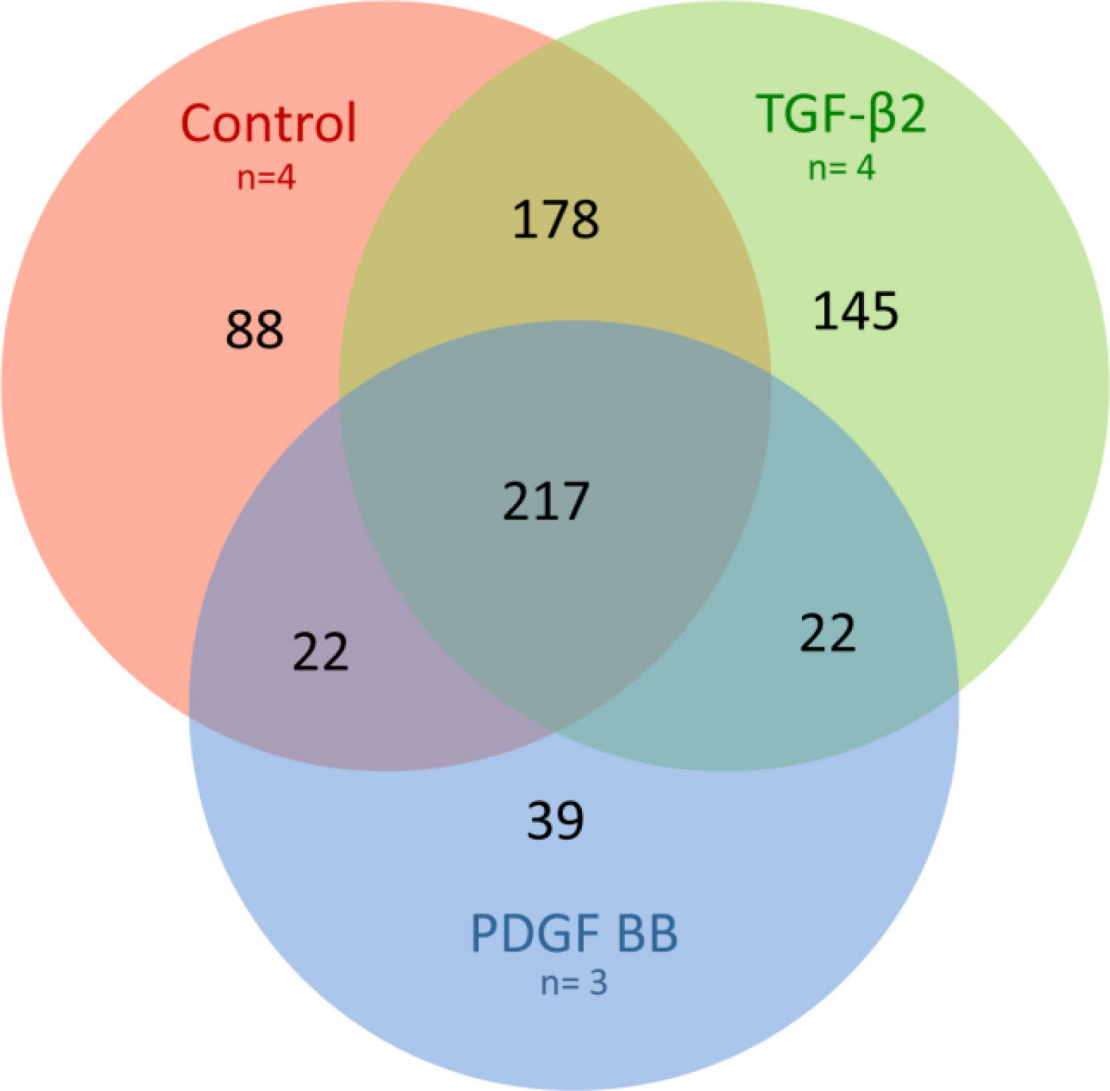
Venn diagram of microarray results. Overlapping circles demonstrating the number of gene transcripts in common, partially in common or unique to each specific exosome treatment group. Of the significant transcripts, 217 were common for all 3 treatment groups, 88 were unique for the control exosomes, 145 unique for the TGF-β2 exosomes and 39 unique for the PDGF-BB exosomes. The control group and TGF-β2-treated group shared 178 transcripts, the TGF-β2-treated group and PDGF-BB-treated group shared 22 transcripts and the PDGF-BB-treated group and control group shared 22 transcripts. Red circle: control exosomes, green circle: TGF-β2-derived exosomes and blue circle: PDGF-BB-derived exosomes.

The gene transcripts with a detection p-value <0.01 (**) were defined as significant. However, for the transcript to be confirmed as present in the exosomes, the transcript also had to be found significant (detection p-value <0.05 and signal intensity >30) in the corresponding cell group. With these settings, 505 transcripts were found in all samples of the control group (n=4), 562 transcripts in all samples of the TGF-β2-treated group (n=4) and 300 in all samples of the PDGF-BB-treated group (n=3) (Supplemental Table 1). Of these transcripts, 217 were common for the 3 groups, 88 were unique for the control exosomes, 145 unique for the TGF-β2 exosomes and 39 unique for the PDGF-BB exosomes (Fig. 4, Supplemental Table 1). The control group and TGF-β2-treated group shared 178 transcripts, the TGF-β2-treated group and PDGF-BB-treated group shared 22 transcripts and the PDGF-BB-treated group and control group shared 22 transcripts (Fig. 4, Supplemental Table 1).

Examples of unique transcripts in the TGF-β2-treated group were NFAT5 and HDAC5.

In the common group of transcripts, found in all 3 exosome treatment groups, about 13% were unknown loci, about 15% were ribosomal mRNAs and about 5% were connected to the energy supply system and the mitochondria.

**Fig. 5:**
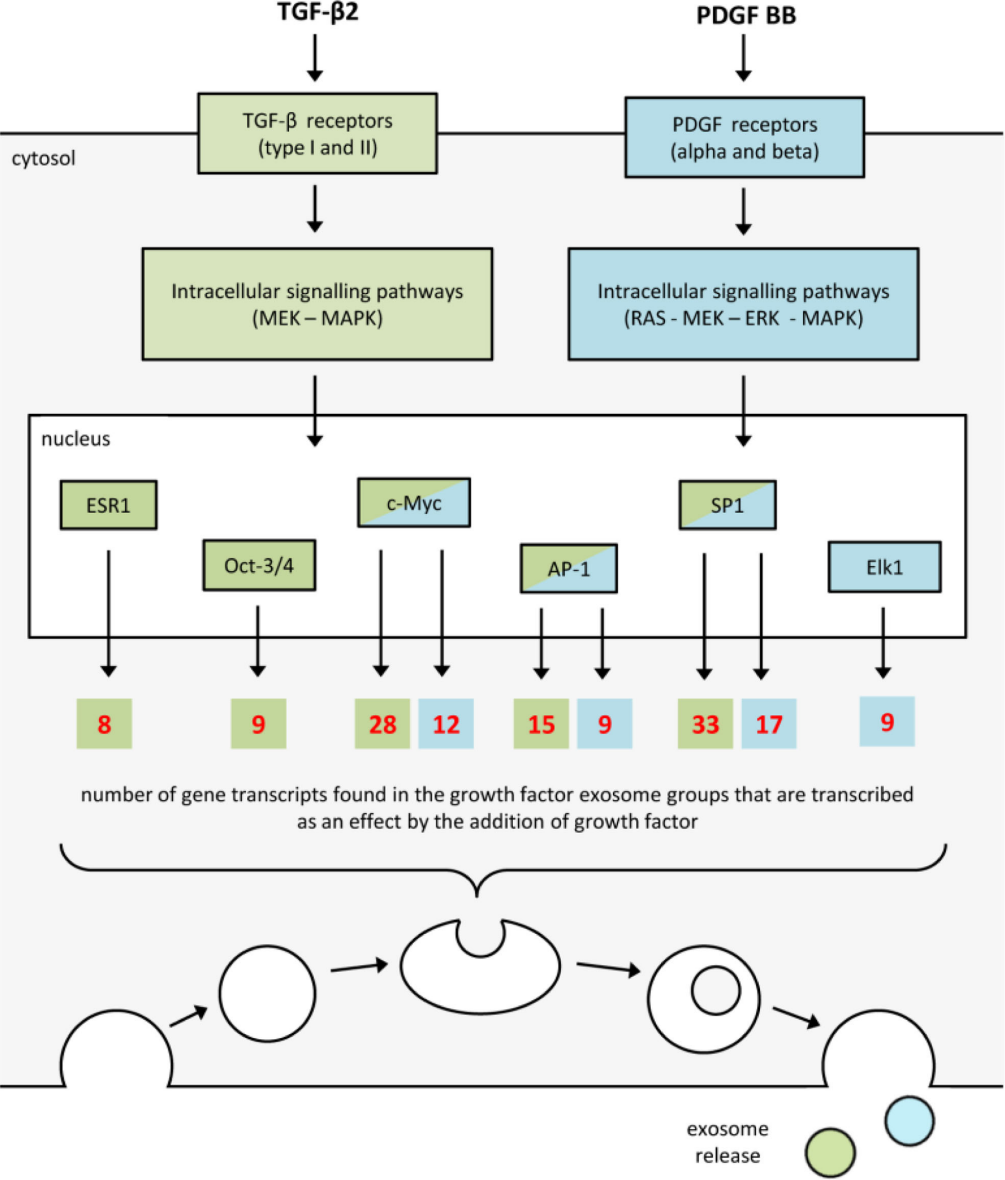
Growth factor signalling explaining the presence of transcripts in the growth-factor-derived exosomes. Analyzing all gene transcripts uniquely found in the growth factor groups with GeneGo MetaCore direct interaction network analysis indicated that the presence of 47.5% of the transcripts found in the TGF-β2-derived exosomes and 36.5% of the transcripts in the PDGF-BB-derived exosomes could be the result of a downstream transcription induced by the growth factor stimulation. Oct-3/4, octamer-binding transcription factor 3/4; ESR1, oestrogen receptor 1; AP-1, activator protein 1; SP1, specificity protein 1; c-Myc, myc proto-oncogene protein; Elk1, E twenty-six (ETS)-like transcription factor 1.

The MetaCore direct interaction network analysis of transcripts specifically found in the exosomes derived from growth factor treated cardiomyocytes revealed that 61 (36.5%) of the transcripts found in the TGF-β2 exosomes and 29 (47.5%) of the transcripts found in the PDGF BB exosomes could be explained as a direct result of the added growth factor. A number of transcription factors were identified to possibly be responsible for the transcription of several of the transcripts specifically found in the exosomes derived from the growth factor stimulated cardiomyocytes (Fig. 5). In the TGF-β2-treated group octamer-binding transcription factor 3/4 (Oct-3/4), oestrogen receptor 1 (ESR1), activator protein 1 (AP-1), specificity protein 1 (SP1) and myc proto-oncogene protein (c-Myc) were most prominent (Fig. 5). In the PDGF-BB-treated group, E twenty-six (ETS)-like transcription factor 1 (Elk1), AP-1, SP1 and c-Myc were most prominent (Fig. 4).

## Discussion

The main finding of the present study was the unequivocal response of cultured cardiomyocytes to the stimulation with 2 different growth factors in terms of transcriptional contents of secreted exosomes. Furthermore, different growth factors induce individual responses.

All gene expression analyses were performed on 4 different samples in each exosome treatment group. Although 1 PDGF-BB exosome sample was considered an outlier and was removed, the number of samples used strengthens the validity of the gene transcripts found in each exosome treatment group. The corresponding cell sample to the outlier in the PDGF-BB exosome group was also an outlier in the microarray analysis of the cellular transcripts.

In the common pool of transcripts, found in all 3 groups (TGF-β2-treated, PDGF-BB-treated and controls), almost 15% of the transcripts were ribosomal and 5% were connected to the energy supply system, which was also seen in our previous characterization study (7). This basic transcriptional package would include structural ribosomal mRNA as well as transcripts coding for proteins involved in the generation of energy in the target cell. One of the characteristics of cardiomyocytes is that they contain a large number of mitochondria, due to the high, constant energy demand. Another feature of the cardiomyocyte is the high turnover of proteins, which could explain the ribosomal mRNA content.

Apart from the common pool of 217 transcripts, there was a group of specific transcripts uniquely found in the control exosomes (88 transcripts), containing a variety of mRNAs with different functions and protein products. Together, these groups would represent the habitual cardiomyocyte state and include transcripts coding for proteins involved in intracellular transport, MAPK-signalling pathways and the nucleus.

The MetaCore direct interaction network analysis indicated that the presence of 36.5% of the transcripts found in the TGF-β2-derived exosomes and 47.5% of the transcripts in the PDGF-BB-derived exosomes could be the result of a downstream transcription induced by the growth factor stimulation.

In the specific group of transcripts for the TGF-β2 group (145 transcripts), nuclear factor of activated T-cells 5 (NFAT5) was found. NFAT5 is involved in cellular proliferation and NFAT5 mRNA expression is particularly high in proliferating cells (30). NFAT5 is a transcription factor that stimulates the expression of hypertrophic genes together with other transcription factors, such as GATA4 and activating protein-1 (31). This result indicates that TGF-β2 affects the exosome content in mediating both a proliferation and hypertrophy signal.

Furthermore, in the group of transcripts that were common for both growth factors (22 transcripts), histone deacetylase 5 (HDAC5) was found. HDAC5 is known to regulate the differentiation of mesodermal cells into cardiac muscle cells (32) but also as a transcriptional regulator repressing the expression of hypertrophic genes (33). This finding, of a signal of both an anti-hypertrophic and a differentiation nature, might indicate that proliferation and cardiac development are favoured before hypertrophic progression in the cell culture, since the cardiomyocytes are proliferating at a high rate in a cultured state.

## Conclusion

To conclude, cardiomyocytes-released exosomes contain a basic package of transcripts common for exosomes derived from unstimulated as well as growth factor stimulated cardiomyocytes. In addition, the growth factor treatment of the cardiomyocytes seems to alter the exosome transcriptional content, mediating signals for proliferation, development and hypertrophy. Accordingly, we conclude that there is a difference in exosomal content between control exosomes and exosomes derived from growth factor stimulated cardiomyocytes.

## References

[1] Keller S, Sanderson MP, Stoeck A, Altevogt P (2006). Exosomes: from biogenesis and secretion to biological function. Immunol Lett.

[2] Simons M, Raposo G (2009). Exosomes – vesicular carriers for intercellular communication. Curr Opin Cell Biol.

[3] Raposo G, Nijman HW, Stoorvogel W, Liejendekker R, Harding CV, Melief CJ (1996). B lymphocytes secrete antigen-presenting vesicles. J Exp Med.

[4] Babiker AA, Nilsson B, Ronquist G, Carlsson L, Ekdahl KN (2005). Transfer of functional prostasomal CD59 of metastatic prostatic cancer cell origin protects cells against complement attack. Prostate.

[5] Valadi H, Ekström K, Bossios A, Sjöstrand M, Lee JJ, Lötvall JO (2007). Exosome-mediated transfer of mRNAs and microRNAs is a novel mechanism of genetic exchange between cells. Nat Cell Biol.

[6] Ronquist GK, Larsson A, Ronquist G, Isaksson A, Hreinsson J, Carlsson L (2011). Prostasomal DNA characterization and transfer into human sperm. Mol Reprod Dev.

[7] Waldenstrom A, Gennebäck N, Hellman U, Ronquist G (2012). Cardiomyocyte microvesicles contain DNA/RNA and convey biological messages to target cells. PLoS One.

[8] Camussi G, Deregibus MC, Bruno S, Cantaluppi V, Biancone L (2010). Exosomes/microvesicles as a mechanism of cell-to-cell communication. Kidney Int.

[9] Blanchard N, Lankar D, Faure F, Regnault A, Dumont C, Raposo G (2002). TCR activation of human T cells induces the production of exosomes bearing the TCR/CD3/zeta complex. J Immunol.

[10] Koga K, Matsumoto K, Akiyoshi T, Kubo M, Yamanaka N, Tasaki A (2005). Purification, characterization and biological significance of tumor-derived exosomes. Anticancer Res.

[11] Llorente A, de Marco MC, Alonso MA (2004). Caveolin-1 and MAL are located on prostasomes secreted by the prostate cancer PC-3 cell line. J Cell Sci.

[12] Wolfers J, Lozier A, Raposo G, Regnault A, Théry C, Masurier C (2001). Tumor-derived exosomes are a source of shared tumor rejection antigens for CTL cross-priming. Nat Med.

[13] Skokos D, Le Panse S, Villa I, Rousselle JC, Peronet R, David B (2001). Mast cell-dependent B and T lymphocyte activation is mediated by the secretion of immunologically active exosomes. J Immunol.

[14] Ronquist G, Hedstrom M (1977). Restoration of detergent-inactivated adenosine triphosphatase activity of human prostatic fluid with concanavalin A. Biochim Biophys Acta.

[15] Faure J, Lachenal G, Court M, Hirrlinger J, Chatellard-Causse C, Blot B (2006). Exosomes are released by cultured cortical neurones. Mol Cell Neurosci.

[16] Gupta S, Knowlton AA (2007). HSP60 trafficking in adult cardiac myocytes: role of the exosomal pathway. Am J Physiol Heart Circ Physiol.

[17] Hellman U, Mörner S, Engström-Laurent A, Samuel JL, Waldenström A (2010). Temporal correlation between transcriptional changes and increased synthesis of hyaluronan in experimental cardiac hypertrophy. Genomics.

[18] Azhar M, Brown K, Gard C, Chen H, Rajan S, Elliott DA (2011). Transforming growth factor beta2 is required for valve remodeling during heart development. Dev Dyn.

[19] Singla DK, Sun B (2005). Transforming growth factor-beta2 enhances differentiation of cardiac myocytes from embryonic stem cells. Biochem Biophys Res Commun.

[20] Azhar M, Schultz Jel J, Grupp I, Dorn GW, Meneton P, Molin DG (2003). Transforming growth factor beta in cardiovascular development and function. Cytokine Growth Factor Rev.

[21] Parker TG, Packer SE, Schneider MD (1990). Peptide growth factors can provoke “fetal” contractile protein gene expression in rat cardiac myocytes. J Clin Invest.

[22] Romano F, Chiarenza C, Palombi F, Filippini A, Padula F, Ziparo E (2006). Platelet-derived growth factor-BB-induced hypertrophy of peritubular smooth muscle cells is mediated by activation of p38 MAP-kinase and of rho-kinase. J Cell Physiol.

[23] Romashkova JA, Makarov SS (1999). NF-kappaB is a target of AKT in anti-apoptotic PDGF signalling. Nature.

[24] Chintalgattu V, Ai D, Langley RR, Zhang J, Bankson JA, Shih TL (2010). Cardiomyocyte PDGFR-β signaling is an essential component of the mouse cardiac response to load-induced stress. J Clin Invest.

[25] Claycomb WC, Lanson NA, Stallworth BS, Egeland DB, Delcarpio JB, Bahinski A (1998). HL-1 cells: a cardiac muscle cell line that contracts and retains phenotypic characteristics of the adult cardiomyocyte. Proc Natl Acad Sci U S A.

[26] Mathivanan S, Simpson RJ (2009). ExoCarta: a compendium of exosomal proteins and RNA. Proteomics.

[27] Banhegyi G, Baumeister P, Benedetti A, Dong D, Fu Y, Lee AS (2007). Endoplasmic reticulum stress. Ann N Y Acad Sci.

[28] Miller JC, Miller JN (1993). Statistics for analytical chemistry.

[29] Kalra H, Simpson RJ, Ji H, Aikawa E, Altevogt P, Askenase P (2012). Vesiclepedia: a compendium for extracellular vesicles with continuous community annotation. PLoS Biol.

[30] Mak MC, Lam KM, Chan PK, Lau YB, Tang WH, Yeung PK (2011). Embryonic lethality in mice lacking the nuclear factor of activated T Cells 5 protein due to impaired cardiac development and function. Plos One.

[31] Rohinia A, Agrawal N, Koyani CN, Singh R (2010). Molecular targets and regulators of cardiachypertrophy. Pharmacol Res.

[32] Karamboulas C, Swedani A, Ward C, Al-Madhoun AS, Wilton S, Boisvenue S (2006). HDAC activity regulates entry of mesoderm cells into the cardiac muscle lineage. J Cell Sci.

[33] Backs J, Olson EN (2006). Control of cardiac growth by histone acetylation/deacetylation. Circ Res.

